# Cardiomegaly Masquerading as a Pediatric Thymoma: A Case Report

**DOI:** 10.7759/cureus.11125

**Published:** 2020-10-24

**Authors:** Matthew R Figlewicz, Rachel E Bridwell, Hannah Beal, Amber Cibrario, Christopher N Belcher

**Affiliations:** 1 Emergency Medicine, Brooke Army Medical Center, Fort Sam Houston, USA; 2 Pediatrics, Brooke Army Medical Center, Fort Sam Houston, USA

**Keywords:** pediatric, thymoma, cardiomegaly, chest pain

## Abstract

Thymoma is a very rare pediatric tumor, accounting for less than 1% of all childhood mediastinal tumors with scant literature, and only 23 pediatric cases were identified by a pediatric tumor surveillance registry between 1973 and 2008. In contrast to adult thymomas, pediatric thymomas have an aggressive tendency, though the majority is discovered as incidental findings. Patient presentations to the emergency department (ED) are often subtle and non-specific such as dyspnea, cough, and chest pain, requiring a broad differential on the part of the emergency clinician. Because of this presentation, diagnosis often occurs later in the disease process when compared with adults. Chest radiograph may demonstrate an enlarged thymic shadow or cardiomegaly, necessitating further cardiac workup, commonly routed through cardiology. Computed tomography and biopsy are required for definitive diagnosis, requiring a multidisciplinary approach to management. We present a case of a 16-year-old female complaining of progressive dyspnea and chest pain over the course of one to two months with radiographic cardiomegaly. She was found to have a Masaoka stage III World Health Organization (WHO) type B3 thymic endothelial neoplasm and underwent surgical resection.

## Introduction

As an exceptionally rare pediatric diagnosis, thymoma of the anterior mediastinum presents a challenging diagnosis in children, with only 23 pediatric cases identified by a pediatric tumor surveillance registry between 1973 and 2008 [1-4]. Though often incidentally found, children can present with non-specific upper respiratory symptoms or chest pain, dyspnea from chest pain, and dyspnea secondary to mass effect on mediastinal structures. As the majority of thymic epithelial neoplasms (TENs) is present in adulthood between the fourth and fifth decades, there are limited data on the treatment and outcomes in the pediatric population [3]. The presence of symptoms, paraneoplastic or autoimmune syndromes, and age less than 10 at the time of diagnosis are associated with poor outcomes and an increased association with malignancy [3-5]. We present a rare case of an advanced pediatric TEN Masaoka stage III World Health Organization (WHO) type B3 in a 16-year-old female.

## Case presentation

**Figure 1:**
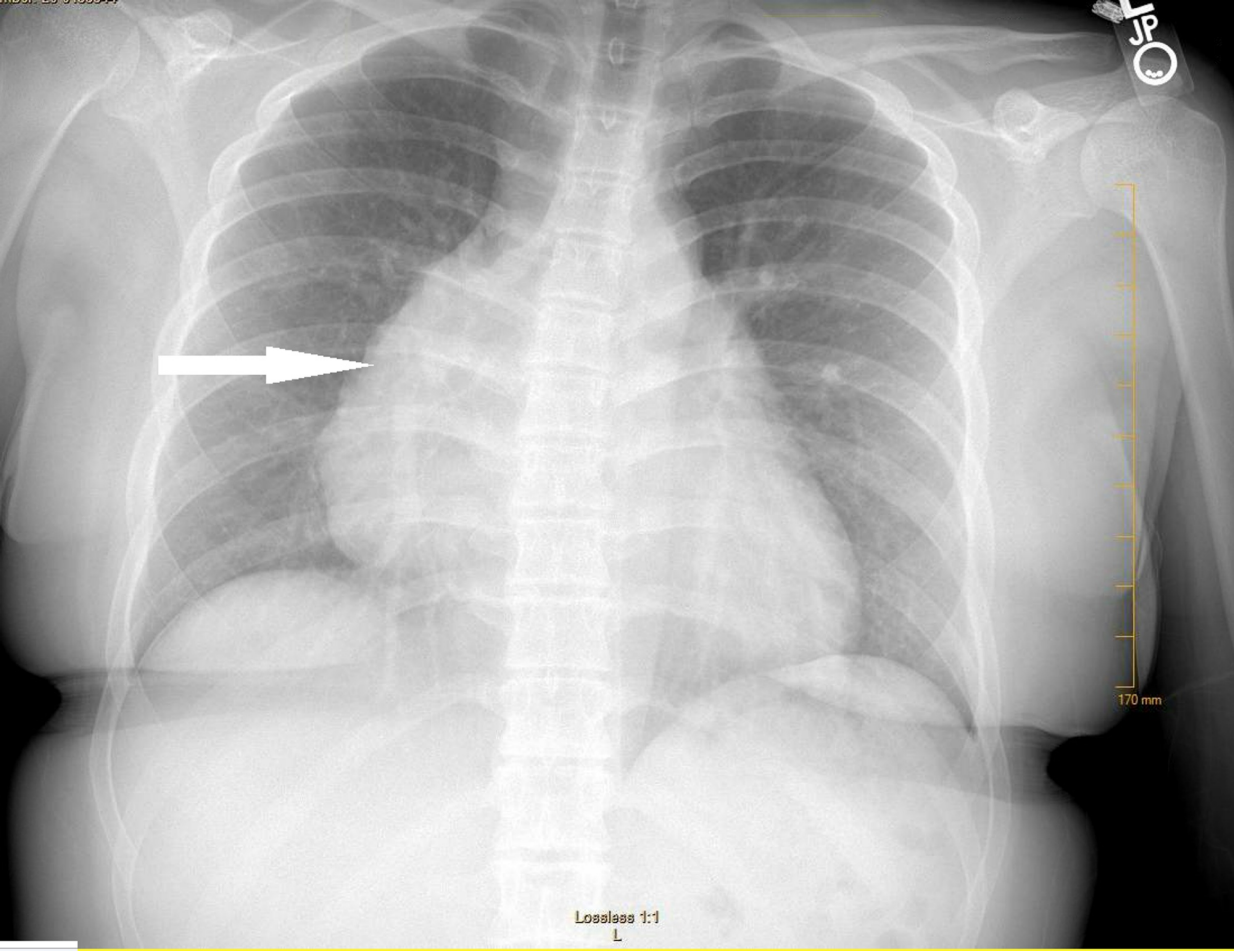
Portable anteroposterior chest x-ray demonstrating cardiomegaly (white arrow) without pleural effusion, pneumothorax, or focal consolidation

A previously healthy 16-year-old female presented to the emergency department (ED) for one day of chest discomfort after playing soccer. She described her upper, compressional in nature localized to upper and central chest, associated with orthopnea and dyspnea. On initial presentation, vital signs were within normal limits. Physical exam was notable for non-reproducible chest pain, and laboratory evaluations including complete blood count, comprehensive metabolic panel, troponin, and brain natriuretic peptide were all within normal limits. Electrocardiogram (ECG) demonstrated normal sinus rhythm at 87 beats per minute. Chest x-ray demonstrated cardiomegaly without focal consolidation, effusion, or pneumothorax (Figure 1).

The patient was discharged with scheduled pediatric cardiology follow-up for cardiomegaly.

**Figure 2:**
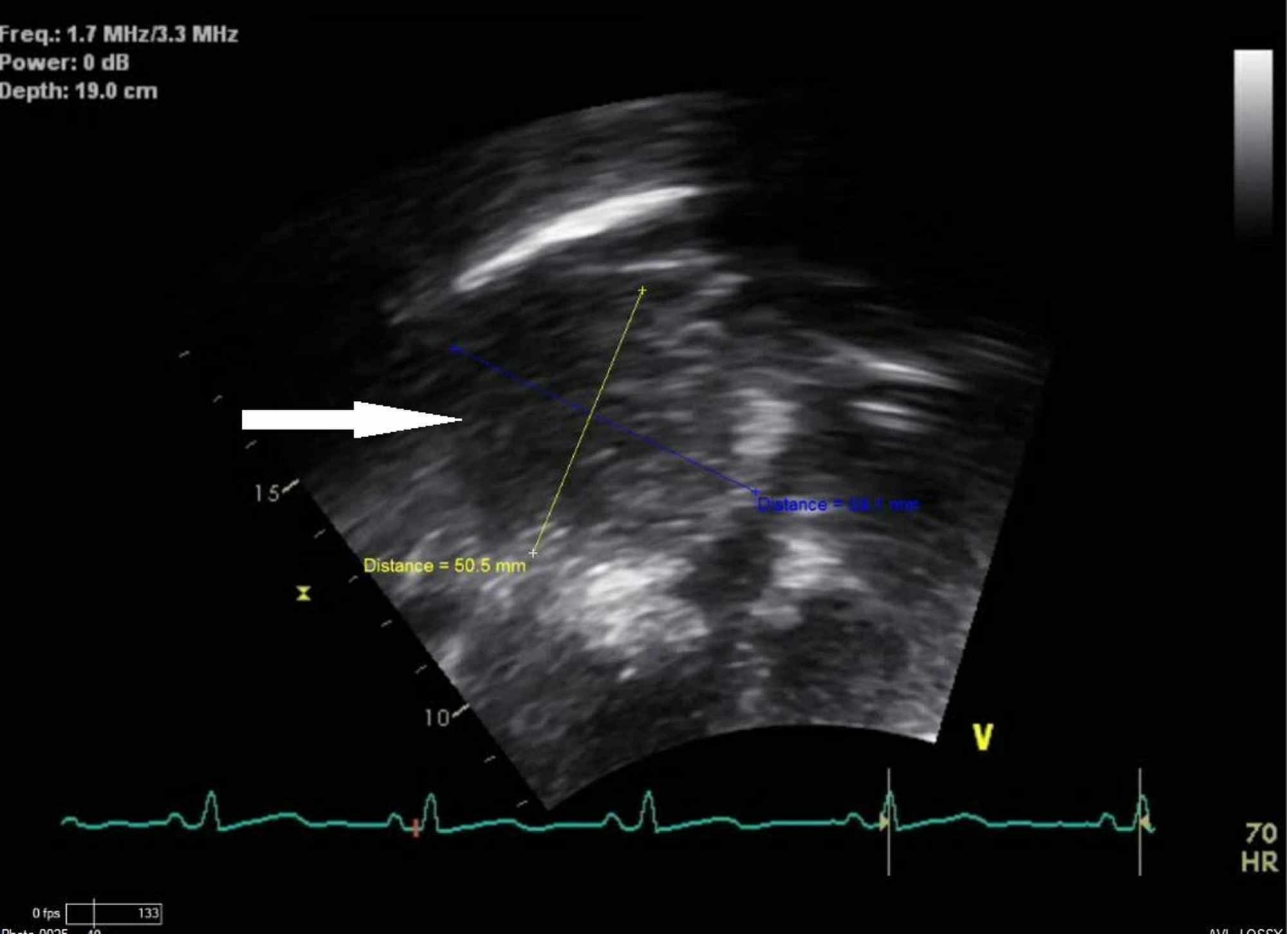
Transthoracic echocardiogram apical four-chamber view still image with homogenous appearing, circular, sessile mass in front of the right atrium (white arrow) measuring 6.1 cm X 8.7 cm in dimension with evidence of right atrial compression

Upon follow-up with cardiology, transthoracic echocardiogram revealed a large circular, sessile, homogenous appearing mass anterior to the right atrium measuring 6.1 cm x 8.7 cm without evidence of internal vascular flow and mild right atrial compression (Figure 2).

**Figure 3:**
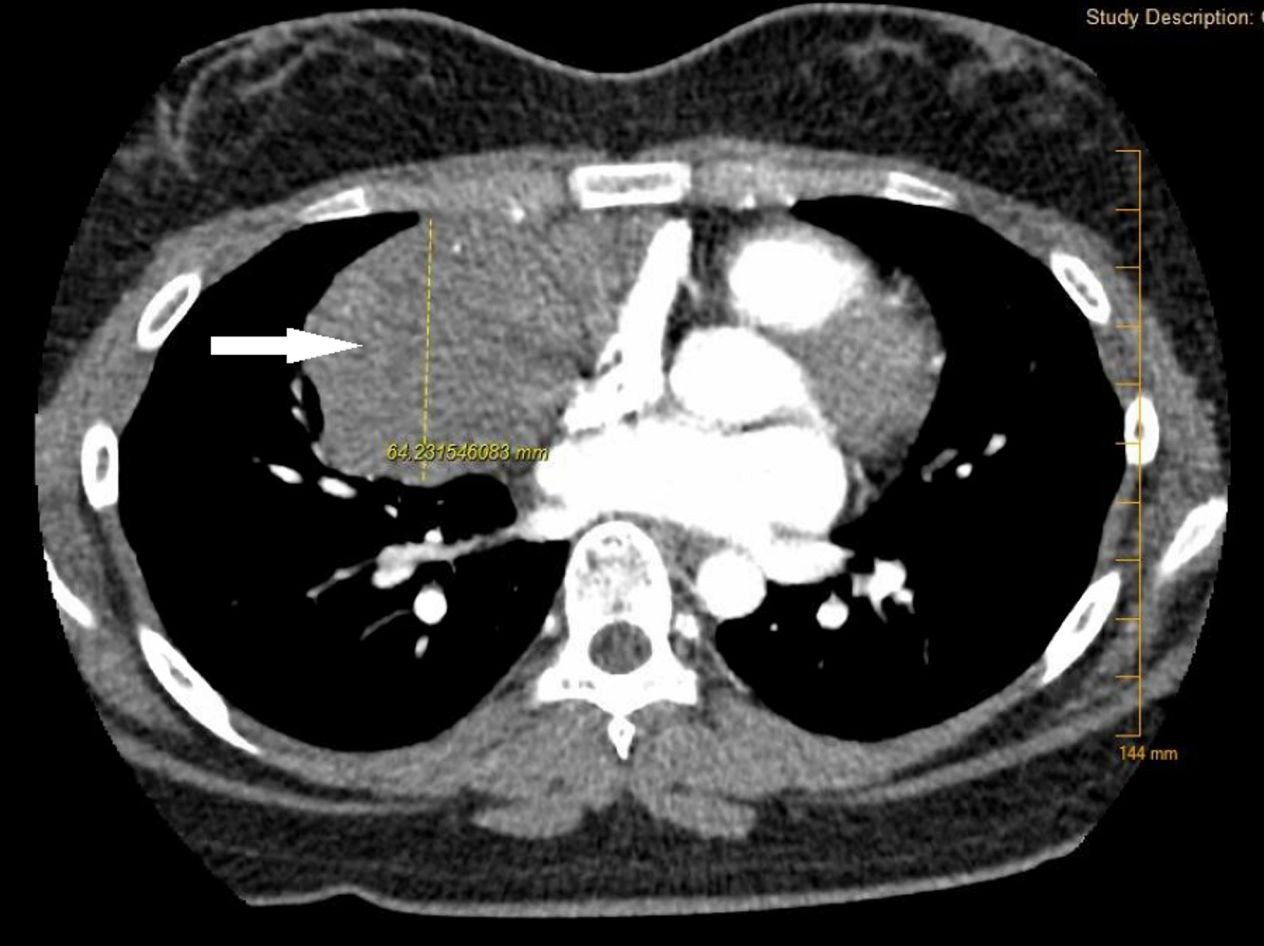
Axial view computed tomography scan of chest with contrast demonstrating 10.7 cm x 4 cm x 10.1 cm mass (white arrow) inseparable from the right heart border pericardium, displacing the heart to the left with associated compression of the superior right atrium and the superior vena cava

**Figure 4:**
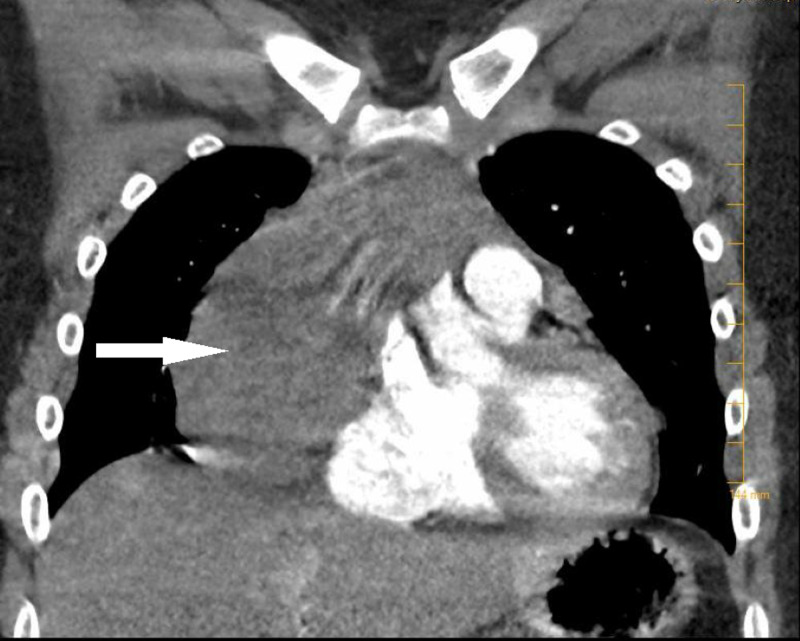
Coronal view computed tomography of chest scan with contrast 10.7 cm craniocaudal x 4 cm anteroposterior x 10.1 cm transverse mass (white arrow) inseparable from the right heart border pericardium, displacing the heart to the left with associated compression of the superior right atrium and the superior vena cava

A computed tomography (CT) chest with contrast was obtained for further evaluation of the mass and revealed a large mass inseparable from the right heart border pericardium, displacing the heart to the left with associated compression of the superior right atrium and the superior vena cava (SVC) (Figures 3, 4).

Needle biopsy revealed a thymoma, Masaoka stage III WHO type B3 (TEN) with atypical presentation. Cardiothoracic surgery successfully excised the tumor, and she was discharged on hospital day eight without complication.

## Discussion

Thymoma in the pediatric population can present in a variety of ways, many of which are non-specific, overlapping with cardiopulmonary pathologies. Presenting symptoms have a wide range from asymptomatic and incidental finding to compressive symptoms of local anatomical structures, including SVC syndrome, respiratory distress, chest pain, cough, orthopnea, and dyspnea [2-4,6]. Patients with stage III-IV masses often present with SVC syndrome, though not present in the above case, contributing to the difficulty of diagnosis in ED [7]. This non-specific presentation should prompt the ED physician to evaluate for potentially life-threatening pediatric causes of chest pain and dyspnea with electrocardiogram and chest radiograph.

Paraneoplastic syndromes or autoimmune disease, most commonly myasthenia gravis, though often associated with adult mediastinal masses and thymomas, are not seen in the pediatric population [2,8]. In contrast to benign tumors, malignant tumors present with lymphadenopathy and B symptoms of fever, weight loss, and night sweats [5]. The presence of these should prompt more extensive evaluation and referral to subspecialists [5]. Younger age at presentation is associated with increased malignancy and worse prognosis [4].

Due to disease rarity, discovery of pediatric thymoma is often an incidental finding on chest radiograph or CT [5]. A contrasted chest CT best evaluates anterior mediastinal masses in the ED; this particular imaging modality allows for differentiation of the mass, location in comparison to local anatomical structures, characterization of the mass, and aid in surgical planning [2,3,5,9]. Referral for biopsy after CT further classifies and stages mediastinal masses, determining the need for adjunctive chemotherapy and further evaluation for metastasis.

The mainstay of treatment is complete surgical resection. In the ED, treatment should be focused on resuscitation and airway management as mass effect may cause airway compromise prompting advanced airway management. Long-term management is extrapolated from the adult population due to low pediatric prevalence [1,2,8,10-11].

## Conclusions

Thymoma is very uncommon in the pediatric population. Its vague and overlapping presentations with other life threats should prompt emergency physicians to keep a high index of suspicion for this disease. These tumors may present with compressive symptoms of surrounding structures or incidentally on imaging and should be fully imaged with a contrasted chest CT. Discovery should prompt a multidisciplinary follow-up to include cardiothoracic surgery, oncology, and cardiology as surgery and adjunctive chemotherapy and radiation are the mainstays of treatment. While rare, this case highlights the uncommon nature of pediatric thymoma in a potentially life-threatening ED presentation.
